# Efficacy of intermittent exposure to bright light for treating maladaptation to night work on a counterclockwise shift work rotation

**DOI:** 10.5271/sjweh.3953

**Published:** 2021-06-29

**Authors:** Heidi M Lammers-van der Holst, James K Wyatt, Todd S Horowitz, John C Wise, Wei Wang, Joseph M Ronda, Jeanne F Duffy, Charles A Czeisler

**Affiliations:** Division of Sleep and Circadian Disorders, Department of Medicine, Brigham and Women’s Hospital and Division of Sleep Medicine, Harvard Medical School, Boston, MA, USA; Current address: Department of Psychiatry and Behavioral Sciences, Rush University Medical Center, Chicago, IL, USA; Current address: Division of Cancer Control and Population Sciences, National Cancer Institute, Rockville, MD, USA

**Keywords:** circadian phase, melatonin, night shift, shift worker, sustained attention

## Abstract

**Objectives:**

Rotating shift work is associated with adverse outcomes due to circadian misalignment, sleep curtailment, work-family conflicts, and other factors. We tested a bright light countermeasure to enhance circadian adaptation on a counterclockwise rotation schedule.

**Methods:**

Twenty-nine adults (aged 20–40 years; 15 women) participated in a 4-week laboratory simulation with weekly counterclockwise transitions from day, to night, to evening, to day shifts. Each week consisted of five 8-hour workdays including psychomotor vigilance tests, two days off, designated 8-hour sleep episodes every day, and an assessment of circadian melatonin secretion. Participants were randomized to a treatment group (N=14), receiving intermittent bright light during work designed to facilitate circadian adaptation, or a control group (N=15) working in indoor light. Adaptation was measured by how much of the melatonin secretion episode overlapped with scheduled sleep timing.

**Results:**

On the last night shift, there was a greater overlap between melatonin secretion and scheduled sleep time in the treatment group [mean 4.90, standard deviation (SD) 2.8 hours] compared to the control group (2.62, SD 2.8 hours; P=0.002), with night shift adaptation strongly influenced by baseline melatonin timing (r^2^= -0.71, P=0.01). While the control group exhibited cognitive deficits on the last night shift, the treatment group’s cognitive deficits on the last night and evening shifts were minimized.

**Conclusions:**

In this laboratory setting, intermittent bright light during work hours enhanced adaptation to night work and subsequent readaptation to evening and day work. Light regimens scheduled to shift circadian timing should be tested in actual shift workers on counterclockwise schedules as a workplace intervention.

Shift work has become increasingly common as our 24/7 global society requires more workers to do their jobs at irregular hours. According to the National Health Interview Survey (NHIS), in 2010 approximately 28.7% of the American workforce was engaged in work outside the standard 09:00–17:00 hours day shift (1). Shift workers are exposed to atypical or irregular sleep-wake schedules, which can lead to misalignment between the endogenous circadian timing system and the sleep–wake cycle. In the short term, this misalignment typically results in poor sleep, increased sleepiness, and performance decrements (2, 3). When prolonged, shift work is associated with increased risk for cardiovascular disease, metabolic syndrome, depression, certain types of cancer, and other health problems (3).

A large variety of round-the-clock work schedules exist, differing by speed and direction of rotation, length of shifts, number of consecutive shifts, and number of shifts per week (4). The magnitude of adverse outcomes varies with the characteristics of the shift work schedule. Counterclockwise shift work schedules (night to evening to day) are associated with worse sleep, lower alertness, and more negative health issues compared to clockwise (day to evening to night) rotations (5–7). While uncommon in Europe, counterclockwise shift rotation schedules are sometimes used in the US (8).

Non-pharmacologic strategies to improve adaptation to shift work based on sleep and circadian principles typically manipulate light exposure patterns and/or sleep timing (9–11). Exposure to light and darkness is the main synchronizer of the circadian timing system, and the use of appropriately timed bright light exposure is an effective approach to enhance circadian adaptation to night shifts (12, 13).

To our knowledge, no study yet has investigated the efficacy of a bright light treatment throughout a complete counterclockwise shift rotation cycle. On such a schedule, not only is the circadian adaptation to night shifts crucial, but equally important is the re-adaptation back to evening and day shifts. Therefore, this laboratory simulation study, carried out between 1995–1998 but not previously reported, aimed to test the hypothesis that a bright light treatment schedule, based on the model of Kronauer and colleagues (14), could rapidly shift circadian rhythms to a night shift schedule as well as readapt them to a day-active schedule during subsequent evening and day shifts. We also tested whether greater circadian adaptation was associated with attenuated performance deficits, determined by performance on a sustained attention task at the end of each shift rotation.

## Methods

### Participants

A total of 29 healthy non-shift working adults, who had a mean age of 27.7 [standard deviation (SD) 6.3] years, (15 women, 14 men) participated in the study. Participants were randomized to the treatment or control group by sex. Fourteen participants (7 women; 7 men) were randomized to receive bright light treatment during work episodes, and 15 participants (8 women; 7 men) were randomized to a control group who were exposed to ordinary levels of room light during work episodes.

Participants were recruited from the community using advertisements in local newspapers and flyers posted on bulletin boards at local colleges and universities. Prior to enrollment, participants were screened, including a physical examination, clinical history, chest radiograph, electrocardiogram, clinical biochemical screening tests of blood and urine, and given either a standardized psychological questionnaire (MMPI) or a structured interview with a clinical psychologist (15). Exclusion criteria included a history of or current significant medical, psychiatric or sleep disorders; history of drug dependency; history of night work; recent (within 3 months) travel across >2 time zones; use of prescription medication. Participants were asked to refrain from using nicotine-containing products, alcohol, caffeine, and all medications for the duration of the study. Each participant had an informed consent meeting and gave written consent prior to beginning the study, which was approved by the Human Research Committee of Partners Health Care and was in accordance with the Helsinki Declaration.

### Study protocol

**Figure 1:**
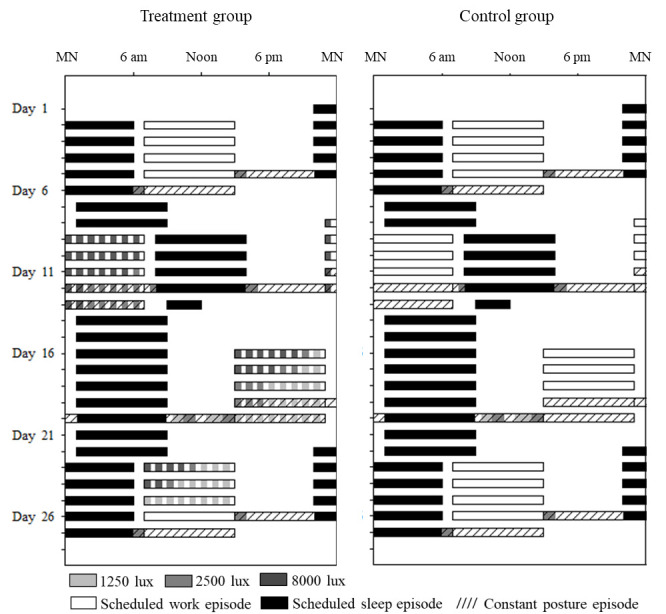
Single raster plots of the 4-week counterclockwise shift work protocols for the treatment and control groups. Clock hour is indicated across the x-axis and study day along the y-axis from top to bottom. Black bars indicate scheduled sleep episodes at home [except during constant postures (CP) where the sleep episodes occurred in the laboratory]. Open bars indicate work shifts under various light exposures (room light 100 lux, 1250 lux, 2500 lux, and 8000 lux). Hashed bars represent CP circadian phase estimation procedures on the 4^th^/5^th^ work shift at the end of each week. Participants were outside the laboratory at all other times.

The 4-week protocol simulated a counterclockwise weekly shift rotation schedule of five 8-hour work days followed by two days off, beginning with day shifts (07:00–15:00 hours), night shifts (23:00–07:00 hours), evening shifts (15:00–23:00 hours), and ending with day shifts (see figure 1). During the 8-hour work episodes in the laboratory, participants remained in study rooms in the Environmental Scheduling Facility at Brigham and Women’s Hospital where they performed four iterations (with rest breaks) of a 1.5-hour computer-based performance battery. After the work episodes, participants left the laboratory. Participants were instructed to adhere to an 8-hour sleep schedule at home, with sleep times specified for each shift (for day shifts, sleep was scheduled from 22:00–06:00 hours, for night shifts from 08:00 to 16:00 hours, for evening shifts from 01:00 to 09:00 hours, and for days off from 01:00 to 09:00 hours). Compliance with the at-home sleep schedule was verified by sleep diaries and wrist activity.

At the end of each work week during the transition from the 4^th^ to the 5^th^ work episode, a 24–32 hour constant posture (CP) regimen took place in the laboratory. After the CP, the participants left the laboratory and had two days off before their next shift type started.

The studies were carried out between 1995 and 1998 but have not been previously reported. The Environmental Scheduling Facility where the studies took place was decommissioned and no longer exists.

### Light exposure

Light was administered from ceiling-mounted fixtures containing 4-foot cool white fluorescent lamps (North American Philips Lighting Corp, Bloomfield, NJ). Light levels were taken in the direction the participant was facing while sitting at the computer desk where they spent most of their shift. In the initial week of day shifts, all participants were exposed to indoor light during work episodes, which was approximately 103 lux in the direction of gaze when sitting at the desk. Control participants worked all shifts in indoor light, whereas the treatment group was exposed to intermittent bright light on the night, evening and day shifts. As indicated in figure 1, intermittent bright light levels of ~8,000, ~2,500 and ~1,250 lux were scheduled based on a mathematical model of the effect of light on the human circadian pacemaker, and consisted of 30 minutes of indoor light alternating with 30 minutes of bright light to initiate a phase delay during the night shifts and a phase advance on the evening and day shifts (14). Details of the timing and intensities of light exposures for the treatment group can be found in the supplementary material (www.sjweh.fi/show_abstract.php?abstract_id=3953).

During the CP, all participants were exposed to 30–60 minutes of ~2500 lux light before and after their work shifts to mimic natural light exposure while commuting during daytime hours. For CP on day shifts, these exposures occurred at 06:00 and 15:00 hours; for night shifts at 07:30 and 16:00 hours; and for evening shifts at 09:00 and 15:00 hours. These are all times at which daylight would be present outdoors in greater Boston during most of the year. In addition, during the initial part of the evening shift CP, daytime light exposure from running errands or exercising outdoors was mimicked in the laboratory by presenting 1250 lux and 2500 lux exposures between 09:30 and 15:00 hours. See figure 1 and Supplement 1. These light “commute time” exposures were not part of the treatment plan produced by the model predictions, but were added to test whether the bright light treatment could overcome light exposure during commute times, which is known to prevent adaptation.

### Constant posture circadian phase assessment

CP were performed at the end of each shift rotation to assess the timing of the circadian rhythm of melatonin secretion relative to the current work and sleep schedule (16). Throughout the 24–32-hour CP, the participant was restricted to a semi-recumbent position in bed. Food and fluid intake were distributed as small hourly snacks. Lights were turned off during the scheduled sleep times, allowing participants to sleep in order to avoid confounding effects of sleep loss on performance for the final work episode of each work week.

Throughout each CP, small blood samples were obtained every 30 minutes via an intravenous forearm catheter connected to a 12-foot IV line. After collection, each blood sample was placed in a Vacutainer tube with EDTA, centrifuged at 2°C for 10 minutes at 2200–2800 rpm, and the resulting plasma was placed in an aliquot tube and frozen at -20°C. Plasma samples were assayed for melatonin shortly after each study was completed using a radioimmunoassay (lower limit of sensitivity 1.1pg/mL; DiagnosTech/Pharmasan Labs, Osceola, WI).

### Sustained attention

To assess sustained vigilant attention, the Psychomotor Vigilance Task (PVT) (17) was taken every two hours beginning 30 minutes after the start of each work shift. The 10-minute PVT was the first scheduled task of a 1.5-hour cognitive test battery which took place 4 times per shift and was scheduled to always occur during times of exposure to indoor light levels in both groups. The PVT required the participant to respond to a visual stimulus appearing on a computer screen by pressing a button with their dominant thumb. The interstimulus interval varied between 2–10 seconds, resulting in ∼100 trials per test. PVT performance has been shown to vary with circadian phase and to decline with duration of sustained wakefulness (18). We report the reaction time (RT) means, medians, and lapses of attention (RT>500ms), as well as reaction time percentile distributions of the PVT taken during each CP.

### Statistical analyses

To examine the timing of melatonin onset, we calculated the time at which plasma melatonin levels rose to 25% of their peak (MEL_25%up_), an established circadian phase marker (16, 19). The peak was determined by fitting a 3-harmonic waveform to the data from the 24-hour baseline (day shift) CP, determining the amplitude of the fitted waveform (maximum-minimum of fitted waveform), and then using linear interpolation between adjacent values to calculate the time at which melatonin levels rose to 25% of this amplitude. This timing method was used to account for the wide variation in the amplitude of plasma melatonin levels between individuals (20). Plasma melatonin offset was defined as the time at which melatonin levels fell to 25% of their fitted peak (MEL_25%down_). The thresholds from the baseline (day shift) CP were used to determine the timing of MEL_25%up/down_ for the remaining CP for each participant. Next, the duration of the melatonin secretory phase for each CP was defined as the interval between MEL_25%up_ and MEL_25%down._

Under normal entrained conditions, participants sleep when their melatonin levels are high with a fitted midpoint of secretion approximately in the middle of the nocturnal sleep episode (21, 22). If the bright light treatment shifted the circadian system appropriately, melatonin should be released during the scheduled sleep time. Therefore, to assess adaptation to the shift schedule, we determined the overlap between the timing of melatonin secretion and the scheduled sleep times (in hours) for each shift schedule. Linear mixed-effects models were applied to study the effects of *group* (control versus treatment) *CP* (baseline, night, evening, and day shift), and their interaction on outcomes, with *participant* as random effect. For each CP, planned post hoc comparisons between the control and treatment groups were performed, where Bonferroni adjustments were used to account for multiple comparisons. Residual plots were checked for model fitting. Correlations between baseline melatonin timing and the degree of adaptation were assessed using Pearson’s correlation coefficient.

PVT mean RT and median RT were recorded in milliseconds (ms). Lapses of attention were defined as RT >500 ms. To calculate reaction time distributions from the PVT, we first computed the 5^th^, 10^th^, 15^th^, 25^th^, 35^th^, 45^th^, 50^th^, 55^th^, 65^th^, 75^th^, 85^th^, 90^th^ and 95^th^ percentiles for the day, night, and evening shift CP for each participant. For each CP, these individual percentile values were then averaged across participants within each group to compute cumulative distributions for the day, night, and evening shift CP for the control and treatment group. We fitted a 4-parameter Weibull function to each average distribution using SAS PROC Reliability, which provided the overall description for each cumulative distribution (23). The *CP* (baseline, night, evening), *group* (control, treatment) and their interaction terms were included in the model. Statistical analyses were performed using SAS 9.4 (SAS Institute, Cary, NC, USA).

## Results

Twenty-nine participants completed the 27-day simulated shift work protocol, which included weekly counter­clockwise shift rotations, for a total of 783 days of study. Complete data on each CP was not available for all participants, but their partial data remained included in the analysis when possible; for details see supplement 2.

### Circadian adaptation

On the baseline (day shift), the melatonin amplitude was similar between the control (mean 37, SD 30 pg/ml) and the treatment group (mean 46, SD 24 pg/ml). The duration of melatonin secretion, the timing of MEL_25%up,_ Midpoint, and MEL_25%down_, and the calculated overlap between melatonin secretion time and scheduled sleep time did not differ between the groups at baseline, as shown in table 1.

**Table 1:** Melatonin timing and phase shift data on each constant posture (CP) for control (C) and treatment (T) groups. Mixed models were carried out to compare melatonin data between the groups. [SD=standard deviation; MEL=melatonin level]

	Baseline		Night shift		Evening shift		Day shift	
			
	C (N=14)	T (N=12)	C (N=13) ^a^	T (N=12)	C (N=14)	T (N=12)	C (N=12)	T (N=11)
	Mean (SD)	Mean (SD)	Mean (SD)	Mean (SD)	Mean (SD)	Mean (SD)	Mean (SD)	Mean (SD)
Time of MEL_25%up_	22:44 (0:34)	22:13 (0:35)	05:10 (3:24)	05:23 (3:56)	01:47 (0:37)	01:15 (1:01)	22:42 (1:24)	22:42 (1:10)
Time of MEL_25%down_	06:33 (0:30)	06:23 (0:25)	11:16 (3:32)	12:52 (3:53)	09:49 (0:31)	09:43 (0:39)	07:05 (0:29)	07:10 (1:15)
Time of melatonin midpoint	02:39 (0:23)	02:18 (0:26)	08:06 (3:27)	09:07 (3:52)	05:48 (0:29)	05:29 (0:42)	03:03 (0:33)	02:56 (1:08)
Melatonin duration (hours)	7.81 (0.7)	8.16 (0.5)	6.09 (1.7)	7.48 ^b^ (1.0)	8.04 (0.6)	8.48 (1.0)	8.38 (1.4)	8.45 (0.8)
Overlap melatonin-sleep (hours)	7.23 (0.6)	7.57 (0.3)	2.62 (2.8)	4.90 ^b^ (2.8)	7.20 (0.6)	7.45 (0.8)	7.01 (0.8)	7.16 (1.0)
Phase shift MEL_25%up_^c^ (hours)			6.42 (3.1)	7.15 (3.7)	3.04 (0.5)	3.02 (0.7)	-0.03 (1.2)	0.48 (0.9)
Phase shift MEL_25%down_^c^ (hours)			4.76 (3.4)	6.47 (3.6)	3.27 (0.6)	3.33 (0.4)	0.64 (0.5)	0.80 (1.1)

a Two subjects showed loss of melatonin amplitude on the night shift, therefore their MEL_25%up_, MEL_25%down_, midpoint, and phase shifts could not be calculated, leaving N=11 for these outcomes.

b Adjusted P≤0.01.

c Phase shift calculations relative to MEL_25%up_ and MEL_25%down_ at baseline.

**Figure 2:**
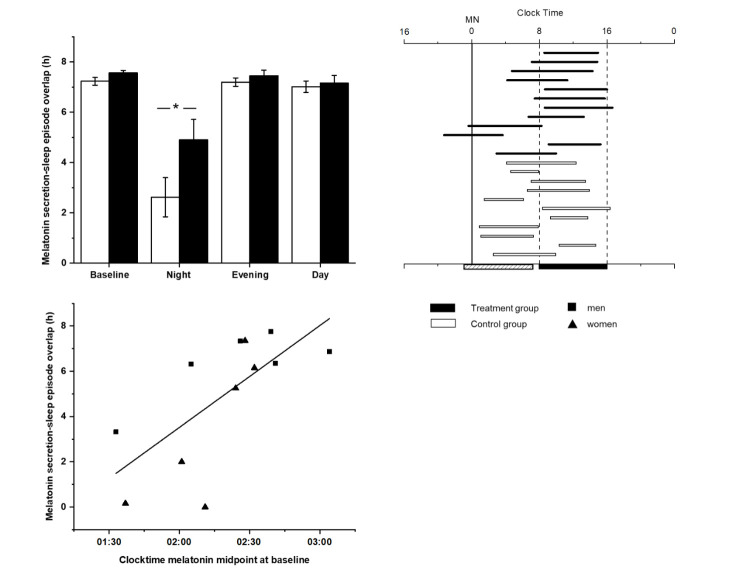
*Panel A:* Average overlap between melatonin secretion and scheduled sleep episode timing (in hours) in the control (open bar) and treatment (black bar) groups for each constant posture (CP) (means ± standard error of means). *Panel B*: Individual melatonin duration (defined as the interval between MEL_25%up_ and MEL_25%down_) in relation to scheduled sleep episode on the night shift CP for control (open horizontal bar) and treatment (black horizontal bar) participants. The vertical dashed bars represent the scheduled sleep episode, which is also depicted as the black filled box on the bottom axis; the hashed bar on the bottom axis represents the work shift. *Panel C:* Clock time of melatonin midpoint at baseline (from the initial day shift) is plotted with respect to the circadian adaptation to the night shift (overlap in hours between melatonin secretion and scheduled sleep) for male (rectangle) and female (triangle) participants in the treatment group; (N=12), r^2^=-0.71, P=0.01).

At the end of the week of night shifts, the MEL_25%up_ and MEL_25%down_ of the control group were shifted 6.4 hours and 4.7 hours later respectively, whereas the MEL_25%up_ and MEL_25%down_ of the treatment group had shifted by 7.2 hours and 6.5 hours. These group differences, along with differences in timing of midpoint, did not reach statistical significance due to large within-group variability (see table 1). In particular, there was a near complete absence of melatonin secretion for the entire 32-hour CP among 2 of the 12 control participants, precluding assessment of their melatonin phase (see supplementary figure S1). When we examined the phase relationship between the timing of melatonin secretion and the scheduled sleep times on the night shift, we found a longer overlap in the treatment group (mean 4.90, SD 2.8 hours) compared to the control group (mean 2.62, SD 2.8 hours; t=-3.72, P=0.002; see figure 2, panel A). The melatonin duration on the night shift was longer for the treatment group compared to the control group (7.48 vs 6.09 respectively; t=-3.430, P=0.005), as can be seen in figure 2, panel B.

On both the evening shift and day shift, no significant differences between the control and treatment groups were found in the melatonin circadian phase markers, the duration of secretion, or the overlap between melatonin secretion and scheduled sleep.

**Figure 3:**
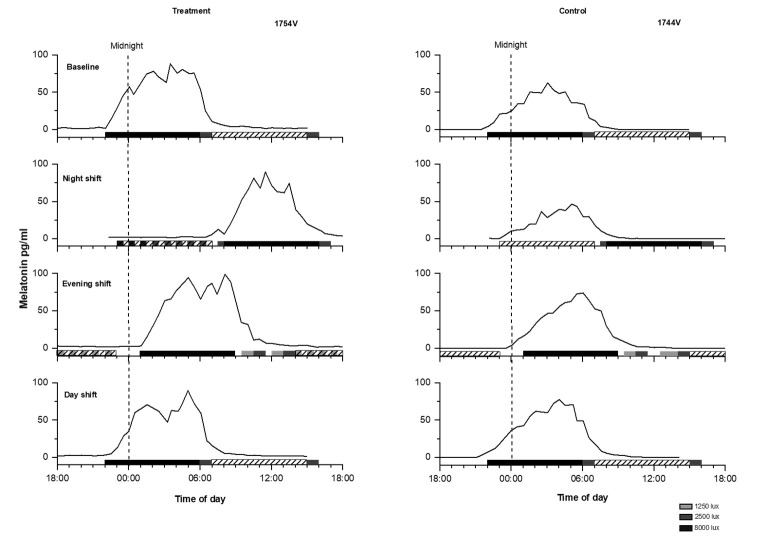
Melatonin profiles for a treatment participant and a control participant. Left panel: Melatonin profile for each constant posture (CP) (baseline, night shift, evening shift, and day shift) of a male treatment participant. Right panel: Melatonin profile for each CP of a female control participant. Black bars represent the scheduled sleep times, diagonal bars represent the work hours and the dashed vertical line represents midnight. During the night shift CP, the control participant showed no overlap in melatonin and sleep timing, whereas the treatment participant did.

On the night shift, the overlap between scheduled sleep and melatonin secretion showed significantly greater variability for both control and treatment participants compared to their overall variability at baseline (night shift SD 3 versus baseline day shift SD 0.5; repeated measures ANOVA F=36.92, P<0.001). Due to this large variability in adaptation to the night shift in both groups, we further explored individual differences in response. Overall, during the night shift the melatonin midpoint occurred during the scheduled sleep time in 8 of 12 treatment participants (67%), whereas for the control participants, the melatonin midpoint occurred during the sleep time in only half of the participants (see figure 2, panel B). In contrast, for the evening shift and day shift, both groups showed 100% overlap between their melatonin midpoint and their scheduled sleep time. As examples, figure 3 shows melatonin profiles in relation to work and sleep times on each CP of a well-adapted treatment participant and a non-adaptive control participant on the night shift.

For the treatment group, we examined whether differences in melatonin timing at baseline (day shift) contributed to the variations in night shift adaptation. We observed a strong relationship between baseline (day shift) melatonin timing and the degree of adaptation on the night shift (r^2^=-0.71, P= 0.01) within the treatment group. The earlier a participant’s melatonin timing at baseline (measured as midpoint of melatonin curve), the less adaptation (ie, hours of overlap between high melatonin secretion and sleep) was shown on the night shift (see figure 2, panel C).

We also explored whether sex differences contributed to the variations in night shift adaptation within the treatment group. The 6 female participants had an average of 3.5 (SD 3.2) hours of overlap between melatonin timing and sleep timing, versus 6.3 (SD 1.6) hours among 6 male participants (Wilcoxon S=28, P=0.09).

### Sustained attention

**Figure 4:**
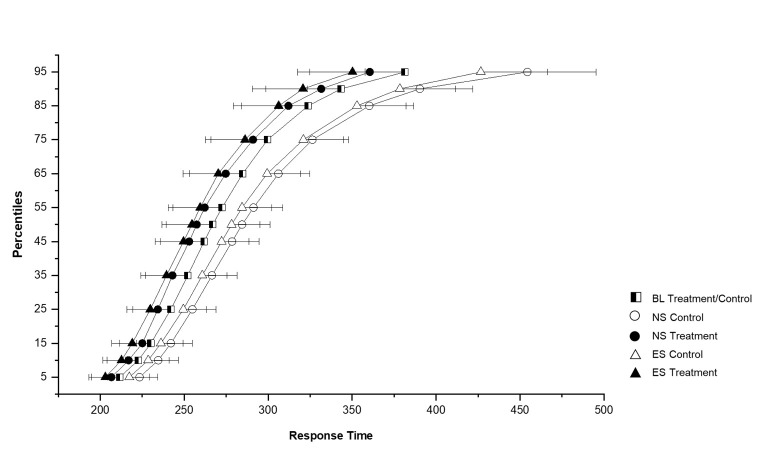
Cumulative response time distributions of the control (N=10) and treatment (N=7) groups on the baseline, night, and evening shift constant postures (CP). The x-axis represents response time in milliseconds. The y-axis represents percentile values of response time. Error bars represent the standard error of the mean. The black/white square symbol represents the combined control and treatment groups at baseline. The control group is represented by open symbols and the treatment group by solid symbols. The night shift is represented by circles and the evening shift by triangles.

There were no significant differences in mean or median RT or number of lapses between the control and treatment groups at baseline or on any other shift, see supplementary table S1. There were no differences in the RT distributions between the control and treatment groups at baseline (see figure 4, presented as combined line). A significant difference in RT distribution was found between the control and treatment group during the night shift (χ^2^_1_=17.68, P<0.001) and the evening shift (χ^2^_1_=14.45, P<0.001). Within the treatment group, there were no differences in the RT distributions between the baseline, night or evening shift, showing that their response times during the night and evening shift remained similar to their response times at baseline. The control group did show a difference in response time distribution between the shift types (χ^2^_2_=8.91, P=0.012). There was a shift to the right in the entire RT distribution for the control group on the night shifts representing cognitive slowing (least mean square comparison vs baseline, P=0.003), and a trend in the same direction on the evening shifts (P=0.07). This is visible in the upper percentile values as shown in figure 4.

## Discussion

We have demonstrated that exposure to bright light can facilitate circadian adaptation to night work and subsequent readaptation to evening and day work among participants scheduled to a counterclockwise shift rotation (ie, rotating weekly from day shift to night shift to evening shift to day shift). While counterclockwise shift rotations are currently not commonly used, they represent one of the most challenging schedules for rotating shift workers. In addition, while there have been numerous studies testing light treatments for circadian adaptation to the night shift, few studies have evaluated whether such treatments impact adaptation to subsequent shift rotations. Here we demonstrated that bright light treatment counteracted neurobehavioral response degradation not only during the night shift but also during subsequent evening work shifts.

To assess how the bright light treatment impacted the circadian system of the participants, our protocol included an assessment of the entire melatonin secretory episode at the end of each shift rotation. We did this based on prior studies showing that circadian interventions can not only shift the timing of the onset of secretion, it can independently affect the offset. Therefore, using only one circadian marker (such as the dim light melatonin onset, DLMO) can give an incomplete view of what happens to the entire melatonin secretion episode. As illustrated in figure 2 panel B, our novel method for evaluating shift work adaptation provides a more comprehensive analyses of the melatonin secretion offset, duration, and phase relationship to scheduled sleep timing.

We designed the light treatment schedule using a mathematical model of human circadian responses to light (14) and found that the light treatment, as predicted, could phase delay shift the circadian rhythm of melatonin secretion from day to night shifts to match the schedule of daytime sleep in the treatment group, despite their off-shift light exposure being uncontrolled. In contrast, participants in the control group showed more erratic responses, with 2.3 hours shorter overlap between melatonin secretion and scheduled sleep than in the treatment group. The finding that the control group still showed 2.6 hours of overlap could be due to the control group being exposed to the same sleep/darkness times as the treatment group. Horowitz and colleagues (12) found that fixed sleep/wake times in darkness alone (without bright light exposure) induced a DLMO phase delay of ~3 hours on the night shift, whereas both fixed sleep/wake times and bright light during the night shift produced a physiological adaptation of ~7 hours on average, similar to what we observed in the treatment group in the present study.

Our results show that an individual’s circadian phase prior to rotation on to the night shift accounted substantially for the variability in the circadian adaptation response to the treatment while on the night shift. This is likely due to the fact that bright light centered after an individual’s body temperature nadir induces a phase advance rather than phase delay shift (24, 25), a limitation that could be overcome by restricting bright light exposure during the second half of the night shift (9, 12). Our finding that earlier melatonin timing was related to less adaptation to night work is consistent with data from field studies that show a negative association between morningness (early chronotype) and the ability to tolerate working at night (26).

Notably, two control participants exhibited no observable melatonin secretion during the entire 32-hour night shift CP, in contrast to their robust nocturnal melatonin secretion during baseline and subsequent CP (see supplementary figure S1). This finding of a loss of amplitude on the night shift was observed in a previous study after a gradual schedule inversion (27). An animal study by Filipski and colleagues (28) showed that a severe amplitude decrease in the corticosterone rhythm (23 to 6 ng/ml) was associated with accelerated tumor growth in SCN-lesioned mice. Furthermore, suppression of melatonin at night has been linked to increased risk for cancer in shift workers (29). More research is needed to understand the phenomenon of temporary loss of melatonin secretion following a schedule inversion in a subset of individuals, to examine whether this loss of melatonin amplitude might also be shown in other circadian rhythms (such as core body temperature or cortisol), and to understand what individual factors may contribute to this loss of amplitude. Much more research is needed to understand whether individuals exhibiting such a loss of melatonin secretion are more or less vulnerable to the adverse health consequences of night shift work. This research may be particularly important for older night shift workers and individuals taking melatonin-suppressing medications such as beta-blockers, many of whom already secret lower amounts of melatonin at night (30).

When the participants made the transition from night to evening shifts, the treatment group had to make a larger adjustment, given the magnitude of adjustment they had made in adapting to the night shift. The finding that the treatment group showed similar melatonin phase timing as the control group on evening shifts, and that both groups showed similar overlap between melatonin secretion and scheduled sleep, demonstrates that the light treatment was successful in re-adaptation by the end of the weekly shift rotation. However, we did not assess adjustment on each individual shift, so we do not know the rate of adjustment to the evening shift that the two groups showed.

Future studies of the impact of light treatments on shiftwork adaptation should focus on sex as a potential relevant factor. Even though our sample size was small, we noticed a trend that the phase-shifting response to the bright light intervention among women was more variable than in men. There are reports that women are more adversely affected by night shifts than men (31, 32). Compelling evidence has shown that there are sex differences in both of the sleep-wake regulatory systems, the circadian system and the sleep-wake homeostat. Compared to men, women tend to be more morning types, have shorter circadian periods (33) and earlier entrained circadian phases (34). These well-defined biological sex differences may make women during the follicular phase of the menstrual cycle more vulnerable to shift work-related sleep loss and circadian misalignment compared to men (35, 36).

Besides enhancing circadian adaptation, the bright light treatment was effective in preventing the slowing of response times on both the night shift as well as on the evening shift. Participants in the treatment group who were exposed to bright light performed similarly on night and evening shifts to day shifts. In contrast, the control group showed significant slowing of their response times on the night and evening shifts, as shown in both laboratory and field studies (23, 37, 38). Our results confirm that adaptation of performance during the night shift can occur in conjunction with circadian adaptation, consistent with previous laboratory studies (39, 40).

Our study was subject to several limitations. First, the circadian phase estimation took place at the end of each work week, and we therefore could not determine the amount of overlap between melatonin secretion and sleep for the initial shifts in each sequence. However, the advantage of our novel approach is that we assessed the entire melatonin secretion curve, including markers of secretion onset and offset (19). This allowed us to test whether there were alterations in the duration of the melatonin secretion episode, not just a change in the timing of melatonin onset (27). Second, we had no information on participants’ light exposure after leaving the laboratory between shifts, and it has been reported that there are individual differences in shift workers’ light exposure after work hours (41), which could have influenced their circadian adaptation. However, by allowing participants to leave the laboratory after each work shift, the bright light treatment had to be able to overcome light exposure during commuting times, a factor recognized to impede adaptation to night work. Third, we assessed circadian adaptation in response to bright light using the rhythm of plasma melatonin, a marker of the central clock located in the suprachiasmatic nucleus (SCN) of the hypothalamus, yet the human circadian system also comprises peripheral clocks found in most tissues and cells (42). While Cuesta and colleagues (43) showed that bright light exposure at night could rapidly reset both the central and peripheral clocks, we did not assess the status of any peripheral clocks. Finally, our study was carried out among non-shift working individuals who were very healthy, not taking medications, and asked to refrain from using caffeine and alcohol during the study. Thus, the generalizability of our results to actual shift workers should be taken with caution, and whether similar phase shifts and performance outcomes would be observed in actual shift workers remains to be tested. A recent field study by Bjorvatn and colleagues (44) found no effect of bright light treatment on subjective and objective sleepiness on night work and consecutive day work among nurses, which may have been due to the participants’ use of medications and inappropriately-timed countermeasures or to inappropriate timing of the light intervention due to changing postures in an active work environment.

Overall though, our findings have important implications for rotating shift workers. They support the importance of acknowledging circadian principles in scheduling work hours (45) and also highlight the importance of taking into account individual circadian timing when applying a countermeasure designed to shift circadian phase. In fact, personalizing working times according to individual circadian timing (ie, chronotype) has been shown to reduce circadian disruption and improve sleep (46). Properly timed light regimens in accordance with individual circadian timing could be an effective workplace intervention for shift workers, improving their on-shift performance, health, and safety.

## Supplementary material

Supplementary material
