# Supplementary material for: Efficacy of intermittent exposure to bright light for treating maladaptation to night work on a counterclockwise shift work rotation
Source: Scand J Work Environ Health. 2021 Jun 29;47(5):356–66. doi: 10.5271/sjweh.3953 (PMC8259707; doi:10.5271/sjweh.3953)
Supplement: Supplementary material [file SJWEH-47-356-S001.pdf]

Efficacy of intermittent exposure to bright light for treating maladaptation to night work on a counterclockwise shift work rotation[1]

by Heidi M Lammers-van der Holst, PhD, James K Wyatt, PhD, Todd S Horowitz, PhD, John C Wise, BS, Wei Wang, PhD, Joseph M Ronda, MS, Jeanne F Duffy, MBA, PhD,[2] Charles A Czeisler, PhD, MD

1. *Supplementary material*
2. *Correspondence to: Jeanne F Duffy, PhD, Division of Sleep and Circadian Disorders, 221 Longwood Avenue, Boston, MA 02115, USA. [E-mail: jduffy@research.bwh.harvard.edu]*

Supplement 1: Details of bright light exposure protocol

Participants randomized to the treatment group were exposed to a complex pattern of intermittent light exposure, consisting of 30 minutes of indoor light (~100 lux) alternating with 30 minutes of brighter light during each eight-hour work episode. The control group worked all shifts in indoor light. The light levels were chosen based on a mathematical model of the effect of light on the human circadian pacemaker (14), and were designed to initiate a rapid phase delay during the night shift week by scheduling the brightest light at the beginning of each shift, then gradually decreasing the duration of the brightest light on each subsequent night shift. For the evening shifts, the lighting was designed to induce a rapid phase advance by administering the brightest light at the beginning of each evening shift (in the afternoon). This phase advance was reinforced for the last week of day shifts by presenting bright light in the morning hours of the first day shifts. The light exposure timing and intensities are outlined as followed:

Week 1 Day shifts

Day 1-4	07:00-15:00 indoor light (~100 lux)

24-h Baseline CP:

Day 5	15:00-16:00 simulated commute (2500 lux)
	16:00-22:00 indoor light
	22:00-06:00 lights out (scheduled sleep opportunity)
Day 6	06:00-07:00 simulated commute (2500 lux)
	07:00-15:00 indoor light

Week 2 Night shifts

Night 1-2	23:00-07:00 8000 lux - indoor light in alternating 30 min intervals
Night 3	23:00-06:00 8000 lux - indoor light in alternating 30 min intervals
	06.00-07:00 2500 lux - indoor light in alternating 30 min intervals

32-h Night shift CP

Night 4	23:00-02:00 8000 lux - indoor light in alternating 30 min intervals
	02:00-07:00 2500 lux - indoor light in alternating 30 min intervals
	07:00-07:30 indoor light
	07:30-08:00 simulated commute (2500 lux)
	08:00-16:00 lights out
	16:00-17:00 simulated commute (2500 lux)
	17:00-23:00 indoor light
Night 5	23:00-02:00 8000 lux - indoor light in alternating 30 min intervals

02:00-07:00 2500 lux - indoor light in alternating 30 min intervals

Week 3 Evening shifts

Evening 1	15:00-21:00 8000 lux - indoor light in alternating 30 min intervals
	21:00-22:00 2500 lux - indoor light in alternating 30 min intervals
	22:00-23:00 1250 lux - indoor light in alternating 30 min intervals
Evening 2	15:00-19:00 8000 lux - indoor light in alternating 30 min intervals
	19:00-21:00 2500 lux - indoor light in alternating 30 min intervals
	21:00-23:00 1250 lux - indoor light in alternating 30 min intervals
Evening 3	15:00-18:00 2500 lux - indoor light in alternating 30 min intervals
	18:00-23:00 1250 lux - indoor light in alternating 30 min intervals

32-h Evening shift CP

Evening 4	15:00-18:00 2500 lux - indoor light in alternating 30 min intervals
	18:00-23:00 1250 lux - indoor light in alternating 30 min intervals
	23:00-01:00 indoor light
	01:00-09:00 lights out
	09:00-09:30 indoor light
	09:30-10:30 simulated daylight (1250 lux)
	10:30-11:30 simulated daylight (2500 lux)
	11:30-12:30 indoor light
	12:30-14:00 simulated daylight (1250 lux)
	14:00-15:00 simulated daylight (2500 lux)
Evening 5	15:00-23:00 1250 lux - indoor light in alternating 30 min intervals

Week 4 Day shifts

Day 1	07:00-11:00 8000 lux - indoor light in alternating 30 min intervals
	11:00-12:00 2500 lux - indoor light in alternating 30 min intervals
	12:00-15:00 1250 lux - indoor light in alternating 30 min intervals
Day 2	07:00-09:00 8000 lux - indoor light in alternating 30 min intervals
	09:00-10:00 2500 lux - indoor light in alternating 30 min intervals
	10:00-15:00 1250 lux - indoor light in alternating 30 min intervals
Day 3	07:00-15:00 1250 lux - indoor light in alternating 30 min intervals

24-h Day shift CP

Day 4	07:00-15:00 indoor light
	15:00-16:00 simulated commute (2500 lux)
	16:00-22:00 indoor light
Day 4-5	22:00-06:00 lights out
Day 5	06:00-07:00 simulated commute (2500 lux)
	07:00-15:00 indoor light

Supplement 2: Missing data

For circadian adaptation, melatonin analyses were performed on 14 (7 men, 7 women) of 15 control participants and 12 (6 men, 6 women) of 14 treatment participants. Three participants were excluded due to insufficient melatonin data because of blood sampling problems. Six participants had missing data on one of the four CP but were included in the analysis for the CP from which they had data. One participant had missing melatonin data from the initial day shift CP, for which we used their final day shift (week 4) melatonin data to determine the threshold to be used to calculate their melatonin phases on the night and evening shift CP. On the night shift CP, two control participants showed a flat melatonin curve, their $MEL_{25\%up/down,}$ phase shifts, and midpoints could not be calculated.

For PVT analyses, we excluded subjects who had incomplete PVT data during either the baseline, night shift, or evening shift CP, leaving a total of N=10 in the control group and N=7 in the treatment group for baseline, night shift and evening shift CP. Data from the last day shift CP were excluded from analysis due to a large number of missing values.

Supplementary figure S1: Melatonin profiles for 2 control participants on each CP. Both participants showed a loss of amplitude during the night shift CP, despite distinct melatonin curves on the baseline, evening, and day shift CP.

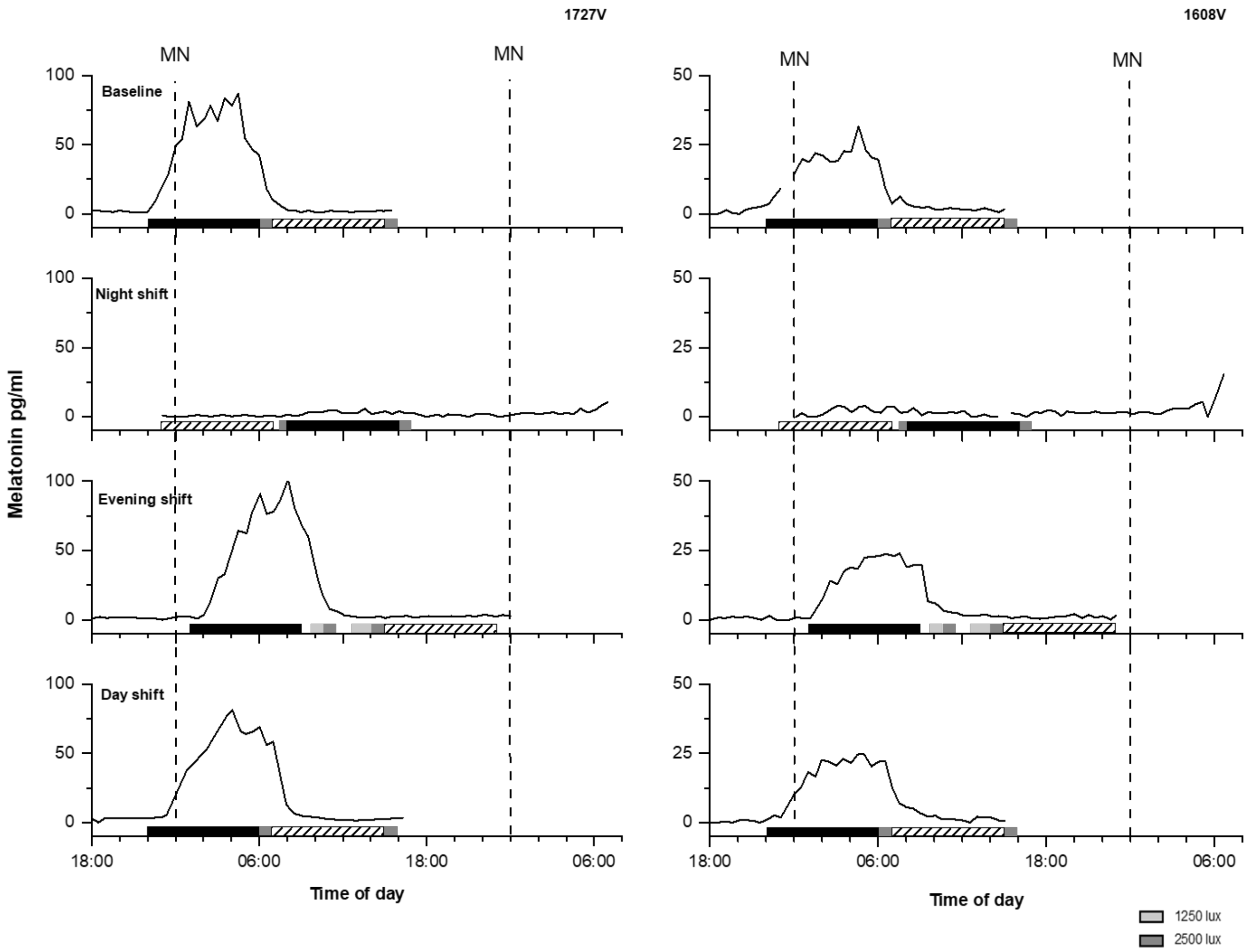

Supplementary table S1: Mean and median reaction time (RT, in milliseconds [ms]), and number of lapses (RT>500 ms) as well as standard deviation (SD) are presented for Psychomotor Vigilance Tests (PVT) from the control and treatment group on each CP. Linear mixed models were applied, with *group* (control, treatment) and *CP* (baseline, night shift, evening shift) as fixed effects, their interaction, and *participant* as a random effect, along with planned post hoc comparisons. N indicates the number of participants whose data contributed to the group. P-values were adjusted for multiple comparisons. No statistically significant differences were found for mean RT, median RT, or number of lapses between the control and treatment groups on any CP.

	Baseline		Night shift		Evening shift	
	C<br>N=10	T<br>N=7	C<br>N=10	T<br>N=7	C<br>N=10	T<br>N=7
PVT-Mean (ms)	286 (54)	273 (60)	312 (80)	269 (54)	298 (68)	266 (52)
PVT-Median (ms)	272 (45)	260 (53)	289 (61)	259 (50)	279 (55)	255 (47)
Lapses	2.3 (4)	1.6 (2)	4.1 (5)	1.6 (3)	3.8 (7)	1.1 (1)
